# Spectroscopic and Microscopic Approaches for Investigating the Dynamic Interactions of Anti-microbial Peptides With Membranes and Cells

**DOI:** 10.3389/fmedt.2020.628552

**Published:** 2021-01-21

**Authors:** Andrew H. A. Clayton

**Affiliations:** Cell Biophysics Laboratory, Optical Sciences Centre, Department of Physics and Astronomy, School of Science, Swinburne University of Technology, Melbourne, VIC, Australia

**Keywords:** anti-microbial, peptide–cell interaction, peptide–peptide interaction, microscopy, fluorescence lifetime imaging (FLIM)-FRET microscopy, spectroscopy

## Abstract

The emergence of microbes resistant to conventional antibiotics is a burgeoning threat to humanity with significant impacts on the health of people and on the health system itself. Antimicrobial peptides (AMPs) hold promise as potential future alternatives to conventional drugs because they form an integral part of the defense systems of other species in the animal, plant, and fungal kingdoms. To aid the design of the next generation of AMPs optimized for human use, we must first understand the mechanism of action of existing AMPs with their targets, ideally in the context of the complex landscape of the living (microbial) cell. Advances in lasers, optics, detectors, fluid dynamics and various probes has enabled the experimentalist to measure the kinetics of molecule–membrane, molecule–molecule, and molecule–cell interactions with increasing spatial and temporal resolution. The purpose of this review is to highlight studies into these dynamic interactions with a view to improving our understanding of AMP mechanisms.

## Introduction

Antimicrobial peptides (AMPs) are part of the innate immune defense system of many organisms in the animal, plant, and fungal kingdoms (1). AMPs found in nature, typically consist of a small number of amino acids (10–30) and are predominantly cationic. The cationic nature of AMPs is thought to facilitate the association with the negatively charged membranes of microbial cells, such as bacteria. Of the 3,000 or so AMPs so far isolated from nature, only a relatively small number of structures have been obtained by high-resolution x-ray or NMR spectroscopy in a membrane environment (2–8). These seminal structural studies have revealed that some AMPs can form pores and/or channels in membranes, disrupting the barrier function of the membrane essential to the life of the cell. Biophysical studies on other classes of AMPs with model membranes have revealed alternative structures where peptides reside on the surface of the membrane, as opposed to passing through it in a transmembrane pore. This has led to alternative membrane-disruption mechanisms, as in the carpet model (9). Variations on pore models (10) or on the carpet model (11), have also been proposed. Some researchers have proposed completely different models altogether (12, 13). A common theme to all the models seems to be some sort of AMP-induced perturbation or disruption of the membrane, either transient, permanent, or both.

How microbes are actually killed by AMPs seems to be less well-understood. Some scientists have proposed different models for cell killing that do not involve pore formation or substantial membrane disruption but may involve peptide translocation and binding to intracellular targets. Bacterial and fungal cells are considerably more complex than phospholipid membranes. End-point assays commonly used in AMP studies with bacteria or fungi can address cell population growth, dead/live cell fractions, or examine cell morphology using methods such as electron microscopy. However, measurements taken at a single time point cannot track intermediate states that may be important in the cell killing mechanism. Studies that dynamically track the interactions of AMPs with targets on or inside cells have the potential to answer this question with a greater degree of confidence.

Here we provide an overview of studies addressing the dynamics of peptide interactions with membranes and cells. Because molecule-molecule, as well as molecule-membrane and molecule-cell interactions are important, we will highlight studies that address the dynamics of these interactions in particular. We will first discuss some key insights gleaned from model membrane studies and then move onto studies conducted on live cells. We conclude with further questions and outlook.

## Model Membrane Studies

Model membranes afford the advantages that the chemical composition and physical phase state can be controlled or varied systematically, and that a large range of physical techniques can be applied to them. Membranes of varying morphology and size can be prepared including vesicles and planar membranes.

The interaction of peptides with membranes is the very first step in a series of processes leading to microbial killing. Studies on model membranes have enabled some of the subsequent steps in the membrane to be elucidated.

The interaction of peptides with membranes can entail a number of structural changes both in the peptide and in the membrane. First, many peptides are unstructured in aqueous solution but can adopt regular secondary structures, such as α-helix or β-sheet when associated with the membrane. Peptide folding, akin to protein folding, is a key dynamic process. Second, peptides usually adopt one of two orientations on/in the membrane. One orientation is with the peptide long axis parallel to the membrane surface. Another common orientation is with the peptide long axis oriented normal to the membrane surface. Orientation changes can be important in processes such as pore formation or peptide translocation through membranes. Third, peptides often associate to form oligomeric complexes or nanoclusters in the membrane. A typical case is pore formation, wherein peptides associate in a transmembrane orientation to form a pore.

There are a few studies aimed at measuring the folding dynamics of monomeric alpha-helices in bilayer membranes. Using a combination of stopped flow fluorescence and stopped flow circular dichroism, Constantinescu and Lafleur (14) detected biphasic kinetics on time scales of tens of milliseconds and seconds for the interaction of the AMP melittin (derived from the sting from a honey Bee), with lipid vesicles. Melittin is a suitable peptide for these studies because in its monomeric form it is predominantly random coil in aqueous solution but adopts an alpha-helix in membranes. The authors showed that the peptide insertion into the bilayer, as measured by quenching of the intrinsic tryptophan fluorescence from the melittin by a brominated-quencher in the bilayer, appeared to precede the full folding of the melittin, as detected by circular dichroism. The authors concluded that changes in apparent folding rates (circular dichroism), promoted by changes in lipid composition, were mainly a reflection of different rates of peptide insertion. These results suggest that insertion and folding are strongly coupled processes, with the former being a rate limiting step.

Tucker et al. (15) used stopped-flow fluorescence spectroscopy to examine the membrane assisted folding of magainin 2, an AMP derived from the skin of an African frog. The authors introduced a special amino acid (p-cyano-phenylalanine) and replaced a phenylalanine with a tryptophan residue to enable detection of coil-helix transition through Forster resonance energy transfer from p-cyano-phenylalanine to tryptophan. The authors detected biphasic fluorescence kinetics with time constants of 10 and 100 ms, attributed to coupled-binding-folding process at the membrane. As for the melittin study mentioned above, the authors here concluded that the measured appearance of the folded helical state is limited by the rate of peptide association with or insertion in the lipid membrane. To explore this aspect further, Tang et al. (16) use a stopped-flow FRET approach with AMP mastaporan X and derivatives with nucleated α-helix formation. The authors concluded that mastaporan associates with the lipid bilayer initially in a non-helical conformation, which rapidly converts into a helical state within the complex anisotropic environment of the lipid bilayer. Taken together, these studies show that while binding and helix formation are coupled events, the determination of the rate of coil-helix transitions in the membrane is difficult owing to the rate limiting peptide-bilayer binding/insertion process. Nevertheless, we can tentatively conclude that the helix formation step is faster than milliseconds and certainly not a rate-limiting process in the overall scheme of AMP activity.

A number of approaches have been employed to examine changes in helix orientation within the lipid bilayer. The pH low insertion peptides (or pHLIPs) developed by Engelman et al. (17) contain hydrophobic residues and have high affinity for membranes at normal or high pH, but at low pH fold and insert across membranes in a transmembrane orientation. Rapid pH modulation provides a means to measure the kinetics of the reorientation step using stopped-flow fluorescence techniques. These studies have revealed two steps in the process, the first being formation of a α-helix at the membrane interface and the second being reorientation of the peptide to form a transmembrane helix. The first step was characterized with a time constant of about 0.1 s, whereas the timescale for the second step ranged from 0.1 to 100 s, dependent on the sequence of the peptide and the composition of the lipid membrane. While not strictly anti-microbial in nature, these model peptides are useful tools for examining helix insertion processes, which are relevant to some AMPs.

Linear dichroism can provide important information on changes in orientation of peptides when associating with lipid bilayers. In one such study, the Rodgers group (18) used cyclic peptides and a combination of circular dichroism and linear dichroism to track changes in peptide secondary structure and peptide orientation during the interaction of peptide with lipid bilayers. After addition of liposomes, the circular dichroism signal indicated a rapid formation of β-structure with no further change throughout the experiment. In contrast, the linear dichroism signal displayed complex kinetics with time constants in the range of seconds, minutes to 100 min. The authors interpreted the changes in LD signal due to rearrangements of the peptide in the lipid bilayer. Interestingly the time-scale for these changes was comparable with the folding of β-barrel outer-membrane proteins suggesting that these model peptides may provide to be good model systems for examining these processes.

The Gai lab (19) used a two probe approach with stopped-flow fluorescence to investigate the binding, insertion and helix–helix association of a peptide (TM anti-αIIb homodimer) known to spontaneously insert and form dimers in bilayers. The complex fluorescence kinetics, observed over a time-scale of milliseconds to seconds, was described by a kinetic model involving peptide binding, insertion, and dimerization within the membrane as well as peptide dimer formation in solution. Interestingly the helix–helix association, to form the homodimer, was the slowest step in the process occurring on a time-scale of seconds and orders of magnitude slower than a diffusion-controlled dimerization process. Thus, the dimerization process itself was the rate-limiting step in this instance.

Anderluh's laboratory (20) used a multi-probe approach to investigate binding, insertion, and oligomerization of a pore-forming α-helical toxin (Equinatoxin II) in membranes. Intrinsic tryptophan fluorescence was used to measure the initial association of the toxin with membranes. NBD fluorescence (from an NBD probe covalently attached to an engineered cysteine derivative) provided insight into the kinetics of helix insertion into the membrane, while AlexaFluor488 fluorescence self-quenching quantitated the extent of helix–helix association (i.e., oligomerization). Impressively, the authors were able to track the kinetics of these fundamental processes in real time, using stopped-flow fluorescence techniques. Protein binding to the membrane was rapid (<1 s), helix insertion took place on the time-scale of seconds, while oligomerization was on the time-scale of tens of seconds. Examination of an engineered protein mutant, which could not insert α-helix into the membrane, bound to the membrane and oligomerized but could not form a functional pore in the membrane. This study brings up an important point that membrane associated proteins and peptides are likely to have multiple states on and in the membrane with the possibility that only some of those states are the biologically active ones (biologically active here means forming a functional pore).

The aforementioned studies have mainly utilized small unilamellar vesicles (either in stopped-flow or flow-aligned (Couette flow) for linear dichroism) as the model membrane system (typical diameter: 50–100 nm). These are very convenient for spectroscopic studies because light scattering is kept to a minimum (relative to studies with cell-sized vesicles) and vesicles survive the shear conditions of rapid fluid mixing or flow. We will next highlight some of the studies performed using giant unilamellar vesicles as the model membrane system. Giant unilamellar vesicles can be prepared to have diameters comparable with the dimensions of biological cells (typical diameter: 1–100 μm) and afford visualization with a microscope so that single objects and single events can be detected.

The Huang laboratory (7) has employed microscopic visualization of melittin action on single giant unilamellar vesicles. Using AlexaFluor488-tagged melittin and calcein-entrapped giant unilamellar vesicles, melittin binding to and calcein release from single vesicles could be observed using fluorescence microscopy. Simultaneous measurements of membrane area was also performed. Melittin binding was observed over a time-scale of 100s s while calcein release was observed only after the majority of melittin was bound to the membrane. In oriented circular dichroism experiments, the authors revealed that at low peptide concentrations melittin forms α-helix aligned parallel to the membrane surface and as the peptide concentration increased, the helices began to align perpendicular to the membrane surface. Lastly, the authors used x-ray diffraction to observe toroidal pores of melittin. Taken together this study revealed that the melittin initially absorbs to the membrane causing an increase in membrane surface area (and thinning of the membrane), this then reduces the energetic cost of forming a transmembrane orientation. Once enough peptide is absorbed to the membrane, the helices then orient to form transmembrane toroidal pores, which allow leakage of large species into the extra-vesicular space. The study here reveals the utility of combining different but related techniques to provide mechanistic information.

Almeida's lab (21) has utilized giant unilamellar vesicles to design useful assays for probing the mechanism of AMP membrane actions and peptide translocations. In one approach, they examined the binding of daptomycin to mixed membranes composed of phosphatidylcholine and phosphatidylglycerol and subsequent effects on the membrane. Using robust colocalization analysis of 1,000s of individual vesicles, they concluded that daptomycin causes formation of daptomycin-phosphatidylgylcerol membrane domains (lipid clusters) in the membrane which eventually cause destabilization and vesicle collapse.

Oreopoulos et al. (22) were able to view membrane domains and phase separation using advanced imaging methods and planar supported lipid bilayers. The authors used atomic microscopy to reveal topographic features of the peptide-induced membrane domains and total-internal reflection fluorescence polarization microscopy to reveal the extent of orientational order of membrane-imbedded probes.

In other series of studies, Almeida's lab developed an assay for examining peptide translocation (23). This assay relies on the creation of vesicles inside giant unilamellar vesicles (vesicles in vesicles). The authors were able to observe peptide and dye influx using confocal microscopy. They were able to identify peptide-assisted influx of dye molecules across giant vesicles, and across the membranes of intra-vesicular vesicles. They observed time scales of influx of the order of tens of minutes (dependent on peptide). They also identified a silent mode of peptide transport, i.e., transport across the giant vesicle and binding to an internal vesicle, which did not cause dye influx. This suggested that a small proportion of the total population of peptides were able to translocate the membrane without causing membrane damage.

Paterson et al. (24) have developed a high throughput giant unilamellar vesicle system suitable for examining the detailed behavior of peptides and used this system to contrast the mechanistic behaviors of melittin and magainin on vesicles with differing membrane compositions. The authors were able to show a spectrum of peptide-induced effects including no effect, burst behavior, pore formation, and carpet-like disruption. The authors interpreted the data in the context of topological and charge effects in the peptides and the lipids, and in terms of a negative feedback model for pore formation. Studies such as these have the potential to provide useful design criteria for the next generation of peptides with enhanced activity and selectivity.

Recently the Huang lab has introduced advances in membrane models and techniques to allow comparison of actions of different anti-microbial agents (25). One of the widely used methods to assess *in vitro* activity of peptides is to use efflux or influx of fluorescent dyes with vesicles. However, the permeability of membranes to dyes is usually time-dependent owing to the time-dependent nature of the peptide-interaction with membranes, making measurements of dye fluxes problematic from conventional fluorescence measurements. Therefore, Huang's team developed a fluorescence recovery after photobleaching protocol to determine dye fluxes through membranes. To compare different AMPs and also to move toward a more realistic membrane system, Huang utilized spheroplasts derived from bacteria. Spheroplasts are created by using antibiotics which cause the bacteria to shed the outer membrane leaving the cytoplasmic membrane. Spheroplasts can also be created to have sizes suitable for observation by microscopy. Using the fluorescence recovery after photobleaching assay revealed that the AMPs known to form pores have similar performance with regard to dye flux in giant unilamellar vesicles as compared with the spheroplasts prepared from bacteria, although differences were noted in the concentration dependence of the phenomena. These conclusions suggest that for the peptides tested, model membranes provide a useful test-bed for pore formation processes occurring at the cytoplasmic membrane.

Wimley's laboratory has been interested in how the performance of peptides as measured by model membrane disruption translates into useful anti-microbial activity against bacteria (26). To this end they designed a peptide library and used a stringent vesicle disruption screen to select the most potent pore forming peptides. They used these peptides of varying membrane disruption abilities to test the hypothesis that better membrane disruption would correlate with improved bacterial sterilization properties. Unfortunately the hypothesis was rejected. The better pore forming peptides were only marginally better in anti-bacterial activity and were more cytotoxic. The authors suggested that the approaches used with vesicles were useful for an initial screen and that conditions that better mimic conditions found in nature are needed in the search for improved AMPs. In the quest for a therapeutic peptide, the Wimley lab have identified a number of barriers to implementation. These barriers include low solubility, degradation by proteases, cell lysis (and associated toxicity), and inhibition due to host cell binding (27).

## Cellular Studies

Leaving the relatively well-studied confines of model membranes we now turn to studies of AMP action on living cells. Imaging and microscopy methods become important in this context since they can reveal the locations, interactions and symptoms of AMPs as they navigate the complex cellular landscape (28).

The Wohland lab (29) utilized single molecule and fluorescence correlation spectroscopy techniques to examine the mechanism of action of quantum-dot labeled a-helical peptide, Sushi-1, on single *Escherichia coli* bacterial cells. The authors identified four steps in the killing mechanism, peptide binding to membrane (as deduced from change in diffusion coefficient of peptide), peptide–peptide association on the membrane (as deduced from decrease in peptide diffusion coefficient as peptide concentration was increased), membrane disruption [without lipopolysaccharide (LPS) detachment] and cytoplasmic leakage through large defects in the membrane [through cytoplasmic Green Fluorescent Protein (GFP) release]. The use of highly sensitive fluorescence detection allowed examination of the peptide behavior at the single molecule level and at very low densities on the membrane. Great care was also taken to ensure that the peptide label did not influence the biological activity of the peptide.

The Weisshaar lab has developed a number of impressive microscope-based assays to examine the mechanisms of AMP actions on single bacteria (30–36). By cleverly combining different genetically encoded and synthetic fluorescence probes, they can track the sequence of events leading to different peptide-induced effects. A particularly ingenious development was the use of a genetically encoded GFP fusion which is trafficked to the periplasmic region of gram negative bacteria. This probe can be used to detect inner membrane disruption, through an increase in the GFP fluorescence from the bacterial cytoplasm. Outer membrane disruption, in contrast, will lead to loss of GFP fluorescence from the bacteria cell entirely. This GFP probe can be combined with other probes, such as cell impermeable nucleic acid-binding dyes, which detect permeability of the cytoplasmic membranes and can be monitored through an increase in fluorescence in the interior of the cell. Probes of other cell states, such as intracellular pH or oxidative stress can also be used. By controlling the delivery of dyes and peptides to the bacteria by means of a microfluidic device, the laboratory can investigate the action of AMPs at different stages of bacterial growth or growth cycle on demand. Microfluidic delivery also allows the use of different concentration profiles of peptides to examine recovery or reversibility of peptide effects. Using these approaches the lab has investigated several peptides including alamethicin, melittin, LL-37, and the cecropin A on *E. coli* cells and *Bacillus subtilis* cells. In the case of the prototypical peptide melittin, the laboratory has shown different features of the membrane disruption mechanism in bacterial cells which involve some combination of changes to outer-membrane permeability (opening and re-sealing and opening again), inner-membrane permeability (opening and re-sealing and opening again) and formation of periplasmic bubbles. The assays developed by the laboratory allowed them to document the ordering and dynamics of these processes, which occurred on the time-scale of minutes to tens of minutes, congruent with the complex interplay of molecular interactions and concentration fluxes over the cellular landscape.

To better understand the nexus between peptide-peptide interactions, as implied in some membrane disruption mechanisms, and cytoplasmic contents release, the Clayton laboratory developed a single-color assay based on fluorescence lifetime imaging microscopy (37). The method relies on the unique lifetime “fingerprints” of fluorescently-tagged peptides in monomeric membrane-bound and oligomerized states, and the lifetime of cytoplasmic GFP (cells) or synthetic fluorophore (membranes), and can track quantitatively the relative amounts of these species in real time. The time-lapse fluorescence lifetime imaging microscopy approach has been applied to a fluorescently tagged melittin derivative upon interaction with single giant unilamellar vesicles, single giant multilamellar vesicles, and *E. coli* bacterial cells (38). Quenching of the lifetime of the fluorescence tag on the melittin peptide was observed over a time scale of minutes, assigned to a progressive increase in peptide–peptide interactions at the membrane. For the unilamellar giant vesicles, once the peptide–peptide interactions reached a threshold level (i.e., once the monomeric and oligomerized peptides were equi-molar at the membrane), release of an intra-vesicular dye occurred rapidly, consistent with pore formation. For the multi-lamellar giant vesicles, dye release was more gradual, which was assigned to the requirement of peptide accumulation at each membrane, pore formation, pore closure and peptide translocation. For the bacterial cells, peptide-peptide interactions increased over time, as for the model membrane systems, but GFP release was not complete, attributed to the complexity of the membranes in the bacteria, and the capacity of the living cell to resist membrane attack. In view of the Weisshaar lab's observations (36), it is likely that the incomplete release may be due to membrane re-sealing events.

AMPs can also transit through cellular membranes without apparent disruption of membrane integrity. Park et al. (39) compared the activities of the AMP burforin II and magainin 2 on *E. coli* cells. Buforin II killed *E. coli* without lysing the cell membrane even at five times minimal inhibitory concentration (MIC) at which buforin II reduced the viable cell numbers by 6 orders of magnitude. However, magainin 2 lysed the cells, resulting in cell death under the same condition. FITC-labeled buforin II was found to penetrate the cell membrane and accumulate inside *E. coli* even below its MIC, whereas FITC-labeled magainin 2 remained outside or on the cell wall even at its MIC. These results are consistent with a mechanism where buforin II translocates across the cell membrane to bind to intracellular DNA and RNA targets.

Shagaghi et al. (40) used a combination of techniques, including fluorescence lifetime imaging microscopy, to address the mechanism of action of a tryptophan-rich peptide thought to act either by pore formation or binding to internal DNA or both. Using a membrane permeant DNA binding dye, which serves as a quencher for FITC fluorescence, the authors detected binding of the puroA-FITC peptide to DNA in the nucleus of a *Candida albicans* yeast cell within 1 min of addition. A propidium iodide assay confirmed that the membranes of the yeast cell were not breached at this point, suggesting that peptide translocation to the nucleus caused only transient membrane damage. About 25–45 min later, puroA-FITC was found to accumulate at the outer membrane of the yeast cell causing a quenching in the lifetime of the FITC fluorescence, due to peptide–peptide interactions (assigned as pore formation) which also coincided with propidium iodide entry into the cells soon afterwards. Changes in cell size, observed at this later step, indicated that the cell integrity was compromised. The kinetics of the pore formation, loss of membrane integrity, shrinkage of cell diameter matched fairly well the kinetics of killing of the cell population indicating that these processes are causally linked. However, the peptide also bound to DNA/nucleic acids (as detected by lifetime quenching) inside the cell before and after membrane disruption and caused cell cycle arrest. Thus, this peptide likely has a dual mode of action involving both intracellular and membrane targets.

A recent study (41) demonstrated the use of fluorescence lifetime imaging microscopy, Fourier transform infrared spectroscopy and atomic force microscopy to examine the interaction of the peptide cecropin D with *E. coli* bacterial cells. Fluorescence lifetime imaging microscopy of FITC-tagged cecropin D revealed a rapid decrease (within minutes) in fluorescence lifetime owing to binding of the peptide to the cells. Fourier infrared spectroscopy revealed changes consistent with peptide interaction with the LPS layer of the outer cell membrane, and a lipid ordering effect. Accordingly, surface topography and nano-mechanical properties were also altered, as revealed by atomic force microscopy, but over a longer time-period of up to an hour.

While single cell assays using microscopic image approaches are clearly powerful at elucidating events at the single cell level, the ultimate goal of an AMP as a therapeutic is to destroy the relevant microbe (fungi or bacteria) at the population level. Some interesting studies have combined single cell with population level assays to examine how peptides function on a “community” level.

Wu and Tan (42), and Snoussi et al. (43) in two separate studies, combined single cell microscopy, population level experiments with mathematical modeling to show that the AMP LL-37 kills a sub-population of bacterial cells, forming dead (or growth arrested) cells, which then absorb the remaining LL-37 from the medium. These authors show that one strain of bacteria can effectively protect another strain of bacteria via this mechanism, although cell-to-cell communication is unlikely to be involved. The results of these two studies reveal the power of combining single cell and population approaches to elucidate emergent behavior on a cell population growth behavior from an AMP. It would be interesting to see if this mechanism applies to other AMPs as well.

Biofilms are often challenging to treat clinically because of resistance to antibiotics, such as daptomycin. In order to investigate potential mechanisms of daptomycin resistance the Steenkeste laboratory (44) created a fluorescent-tagged daptomycin analog and exploited dynamic imaging techniques to examine daptomycin transport within the biofilms. Using time-lapse fluorescence (to investigate daptomycin uptake) and fluorescence recovery after photobleaching (to investigate transport), the authors revealed that the biofilm did not significantly impede daptomycin uptake and diffusion, ruling out the biofilm as a barrier to drug penetration. The authors suggested alternative resistance mechanisms relating to changes in the membranes of the cells due to the altered environment of the biofilm. In another study, the same laboratory (45) investigated the dynamic interactions of vanamycin with planktonic cells and biofilms using fluorescence correlation spectroscopy, fluorescence lifetime imaging microscopy, and fluorescence recovery after photobleaching. The fluorescence correlation spectroscopy method enabled the laboratory to extend their transport/diffusion measurements to lower concentrations of antibiotic while the fluorescence lifetime imaging provided information on changes to molecular environment around the antibiotic. The authors were able to observe changes in the diffusion of vanamycin in the biofilm as compared planktonic cells. However, the authors suggested that the altered diffusion was not ultimately responsible for antibiotic resistance. The techniques developed in these papers could be potentially useful in assessing accessibility and transport of AMPs in biofilms as well.

## Concluding Remarks

In this focused review, I have attempted to provide a flavor of the range of dynamics AMPs undergo when presented with membranes, cells and cell populations. Although these peptides are seemly simple, since they possess no tertiary structure, they are able to provide effective defenses against microbes encountered by many species in the animal and plant kingdoms. Optical spectroscopy and microscopy approaches combined with judicious labeling of peptides, membranes, and sub-cellular structures has enabled the determination of key events leading to cell killing activities. One of the broad results of these studies is that the dynamics detected in cells (by microscopy) appears to be orders of magnitude slower than processes studied in model membranes (by stopped flow fluorescence) and often exhibit lag-phases in behavior for minutes or hours. Peptide binding, secondary structure formation, orientation changes and peptide-peptide interactions in membranes, as studied by rapid mixing techniques occurs on the millisecond to second timescale, which likely reflect initial effects in one leaflet of a lipid bilayer. However, in cells, peptides need to transverse multiple barriers and can undergo a number of subsequent steps in addition to binding, conformation change and peptide-peptide interaction, including peptide nucleation/accumulation, membrane defect/pore creation and defect/pore re-sealing, peptide desorption, peptide translocation, membrane re-binding, peptide transport and interaction with non-membrane targets including (but not limited to) nucleic acids and DNA. The cellular landscape is heterogeneous in several respects (chemically, biologically, spatially, and temporally) and peptide concentrations within and between cells can be highly heterogeneous adding to the observed complexities in dynamics. Moreover, it needs to be remembered that cells are living, social entities, with feedback mechanisms and adaption, which have evolved over billions of years. What is needed are methods to track the physico-chemical states of the peptides in parallel with the relevant biological states of the microbe being targeted. In this way the relevant physical state of the peptide (structure, orientation, oligomeric state, cell location) which impedes microbial action can be better elucidated. It is emerging that membrane permeability may not be the only symptom of AMP action and a greater exploration of peptide-induced cell insults is needed. Improvements in imaging methods and probes will greatly assist in this endeavor.

## Author Contributions

AC wrote the paper.

## Conflict of Interest

The author declares that the research was conducted in the absence of any commercial or financial relationships that could be construed as a potential conflict of interest.
